# Patterns of late gadolinium enhancement in Duchenne muscular dystrophy carriers

**DOI:** 10.1186/1532-429X-16-45

**Published:** 2014-07-09

**Authors:** Vincenzo Giglio, Paolo Emilio Puddu, Giovanni Camastra, Stefano Sbarbati, Sabino Walter Della Sala, Alessandra Ferlini, Francesca Gualandi, Enzo Ricci, Federico Sciarra, Gerardo Ansalone, Marco Di Gennaro

**Affiliations:** 1 Center for Neuromuscular Disease, Uildm, Prospero Santacroce St. 5, Rome 00167, Italy; 2 Cardiology Division and ICU, Ospedale San Paolo, Civitavecchia, Rome Italy; 3 Department of Cardiovascular, Laboratory of Biotechnologies Applied to Cardiovascular Diseases, Respiratory, Nephrological, Anesthesiological and Geriatric Sciences, Sapienza, University of Rome, Rome Italy; 4 Cardiology Division and ICU, Ospedale Madre Giuseppina Vannini, Rome Italy; 5 Radiology Department, Ospedale Madre Giuseppina Vannini, Rome Italy; 6 Department of Medical Science, Section of Medical Genetics, University of Ferrara, Ferrara Italy; 7 Neurology Institute, Catholic University, Rome Italy

**Keywords:** Duchenne muscular dystrophy carriers, Cardiovascular magnetic resonance, Genetics

## Abstract

**Background:**

This study was designed to assess whether cardiovascular magnetic resonance imaging (CMR) in Duchenne muscular dystrophy carriers (DMDc) may index any cell milieu elements of LV dysfunction and whether this cardiac phenotype may be related to genotype. The null hypothesis was that myocardial fibrosis, assessed by late gadolinium enhancement (LGE), might be similarly accounted for in DMDc and gender and age-matched controls.

**Methods:**

Thirty DMDc patients had CMR and genotyping with 37 gender and age-matched controls. Systolic and diastolic LV function was assessed by 2D-echocardiography.

**Results:**

Absolute and percent LGE were higher in muscular symptomatic (sym) than asymptomatic (asy) DMDc (1.77 ± 0.27 vs 0.76 ± 0.17 ml; F = 19.6, p < 0.0001 and 1.86 ± 0.26% vs 0.68 ± 0.17%, F = 22.1, p < 0.0001, respectively). There was no correlation between LGE and age. LGE was seen most frequently in segments 5 and 6; segment 5 was involved in all asy-DMDc. Subepicardial LGE predominated, compared to the mid-myocardial one (11 out of 14 DMDc). LGE was absent in the subendocardium. No correlations were seen between genotyping (type of mutation, gene region and protein domain), confined to the exon’s study, and cardiac phenotype.

**Conclusions:**

A typical myocardial LGE-pattern location (LV segments 5 and 6) was a common finding in DMDc. LGE was more frequently subepicardial plus midmyocardial in sym-DMDc, with normal LV systolic and diastolic function. No genotype-phenothype correlation was found.

## Background

Xp21-linked Duchenne muscular dystrophy carrier (DMDc) status is characterized by skeletal muscle weakness ranging from absence of muscular symptoms to mild or even rapidly progressive Duchenne-like muscular dystrophy. The muscle disease may be associated with cardiac involvement, from no symptoms to overt dilated cardiomyopathy (DCM). It is well established that a minority of carriers is more likely to develop early severe DCM [1] which may appear even in childhood [2]. Thus DCM may become the only limiting factor in DMDc and the first cause of death in these patients [3-5] whose last option for survival is heart transplantation [6-8].

Myocardial fibrosis is best detected and quantified by cardiovascular magnetic resonance (CMR) [9] both in ischemic [10] and nonischemic heart diseases [11] and may be accurately detected by late gadolinium enhancement (LGE). However, LGE studies were undertaken in DMDc quite rarely and few case reports exist [12,13].

The present study was aimed at investigating a large consecutive series of DMDc, proven by DNA analysis and undergoing LGE. The null hypothesis was that myocardial fibrosis, assessed by LGE, might be similarly accounted for in DMDc and gender and age-matched controls. We investigated: a) the proportion of DMDc with myocardial LGE and cardiac involvement and b) the cardiac genotype-phenotype relationships if any.

## Methods

### Study population

We enrolled 30 consecutive female DMDc aged 11 to 63 years (mean age 36 ± 5 years) followed at the Center for Neuromuscular Diseases (Uildm) of Rome, Italy and 37 age-matched healthy female controls (mean age 34 ± 5 years, p = NS). Controls had no cardiac symptoms, history of cardiomyopathy or skeletal muscle disorders and presented with normal electrocardiography (ECG), 2D-echocardiography and CMR. No drug was given to DMDc and controls. Creatine kinase levels were screened in the control group and in all DMDc. The study was approved by the Ethical Board of the Catholic University of Rome. Written informed consent was obtained from all patients and controls. Exclusion criteria were left ventricular (LV) systolic and diastolic dysfunction, diabetes, hypertension, ECG changes suggestive of ischemic heart disease, atrial flutter/fibrillation, any degree of atrioventricular block, valvular heart disease, and LV hypertrophy.

### Clinical evaluation

DMDc and controls underwent physical examination and surface 12-lead ECG. DMDc, none with cardiac symptoms, were classified regarding muscle involvement as: (1) asymptomatic (asy), when characterized by the presence of high CK levels and/or minor myopathic signs like muscle cramps and myalgia, without muscle weakness and (2) symptomatic (sym), when presenting variable degrees of muscle weakness [14]. The degree of skeletal muscle involvement was assessed by a neurologist and all DMDc were divided into two functional groups: a) 21 asy (63%) and b) 9 sym (27%). Of the 9 sym-DMDc, 3 were mildly affected, showing some impairment in running and jumping, 5 moderately affected, with clear strength deficits in specific muscle districts and 1 severely affected and wheelchair-bound. In asy-DMDc, significant coronary artery disease was excluded by negative treadmill exercise testing. In sym-DMDc, myocardial perfusion was assessed by adenosine thallium-201 myocardial perfusion scintigraphy (ATl-201-MPS), according to the guidelines for the clinical use of cardiac radionuclide imaging [15]. ATl-201-MPS was performed in these patients since they could not perform treadmill exercise testing.

### Echocardiography

Conventional 2D-echocardiography was performed by the same operator (VG) in all patients, using a SONOS 5500 (Philips Andover, Mass, US). LV systolic function was assessed by ejection fraction (EF) from LV volumes (derived using the modified Simpson’s rule). LV systolic dysfunction was defined as LVEF ≤55%. LV diastolic function was assessed by Doppler analysis as previously reported [16].

### Dystrophin gene and protein analysis

Extensive molecular analysis was performed in all DMDc. Mutation detection was carried out using Multiplex-Ligation dependent Probe Amplification (MLPA) (deletions and duplications) or sequencing (small mutations), accordingly to the DMD guidelines [17]. Although muscle biopsy may be used for establishing the carrier condition, it is well known that approximately 60% of carriers do not show muscle abnormalities [18].

### Cardiovascular magnetic resonance

CMR was performed using a 1.5-T MR system (INTERA, Philips Medical Systems, Best, the Netherland) with a cardiac 5-element phased-array receiver coil . All images were acquired with ECG-gating, breath-hold steady-state free precession (SSFP) cine sequence for functional analysis, in contiguous short-axis view (10-mm intervals, interslice gap 2 mm, slice thickness 8 mm in plane-resolution 1.2 × 1.8 mm) from the mitral annulus to the apex and 3 long-axis planes, with the patient in a supine position. To assess the contribution of cardiac edema, we performed a T2-weighted segmented triple inversion recovery (T2-wSTIR) imaging module, in 3 short axis slices (8 mm, flip angle 90°, repetition times 2 RR intervals) at the base, mid, and apex and a single long axis-slice in a 4 chamber view, using for imaging a functional surface coil intensity correction. All patients underwent an LGE imaging protocol (repetition time 4.5 ms, echo time 1.7 ms, inversion time 200 to 300 ms) for myocardial scar using a segmented Inversion Recovery-Gradient Echo (IRGE) sequence, adjusting the inversion time and nulling the signal of normal myocardium. Contrast CMR images were acquired on average 10 to 15 min after injection of cumulative 0.1 mmol/kg gadolinium DTPA (Magnevist, Gd-DTPA, Shering AG). Images were obtained in 8 to 14 short-axis and 3 radial long-axis planes. Myocardial enhancement on LGE images was assessed visually and considered positive to a signal-intensity threshold of >2 SD above the mean intensity of a remote reference region [19] and interpreted as present or absent by the consensus of two cardiac CMR-trained physicians. LGE quantity was quantified using manual planimetry, summing the LGE positive areas yielding a total volume (ml). LGE percentage was obtained dividing the total LGE volume by the LV mass. The LGE location and wall motion abnormalities was classified according to AHA for heart imaging [20]. Height and weight were measured in DMDc and controls on the day of scanning; values for volumes and mass were indexed by body surface area (BSA). The Simpson’s method was applied to determine myocardial mass, end-diastolic volume, end-systolic volume, right ventricular (RV) and LV EF by a dedicated software, manually tracing the endocardial and epicardial borders in each short axis slice. Depressed RV and LV systolic function were defined according to the reference values for age and gender [21]. The ventricular volumes, RV and LV function and extent of contrast enhancement, were analysed off-line on a dedicated workstation (Extended MR WorkSpace Release 2.6.3.2, Philips Medical Systems, Best, the Netherland) by 2 CMR-experienced operators blinded to clinical DMDc status.

### Statistics

After testing for normal distribution based on standard parameters, intergroup differences and relations were compared by analysis of variance (ANOVA), correlation matrices and/or unpaired Wilcoxon t-test. NCSS software version 2007 (http://www.ncss.com) was used. A value of p < 0.05 was considered statistically significant.

## Results

Table 1 summarizes the clinical characteristics of the study population. All DMDc had increased values of serum creatine kinase that were normal in the control group (<190 U/L). The ECG was normal in the control group and normal or nearly normal (aspecific T-wave changes), both in asymptomatic and symptomatic DMDc, independent from the LGE presence. Both treadmill exercise testing, and ATl-201-MPS studies were negative. CMR was performed in all DMDc and controls.

**Table 1:** Clinical data in asymptomatic and symptomatic (from the muscular point of view) DMD carriers

**Patient**	**Age (y)**	**Genotype**	**CPK (UI/l)**
Asymptomatic			
1	57	Del ex 46-51	2750
2	21	3-17	840
3	34	Dup ex 5-6-7	1250
4	11	Del Prom + ex 1	2370
5	32	51	4000
6	30	45-52	980
7	47	3-17	1150
8	41	7-25	545
9	46	52	1745
10	37	49-50	3265
11	44	45-50	358
12	34	51	720
13	33	52	2438
14	42	Del Prom	1845
15	42	Leu 2225 Stop	3850
16	37	Dup ex 2	1645
17	44	52-54	2560
18	39	45-50	2745
19	30	51-54	650
20	38	65	3200
21	30	47-54	4250
Symptomatic			
1	20	49-50	3840
2	17	48-54	5770
3	35	45-50	920
4	45	45-52	575
5	49	56	1930
6	63	48-52	4275
7	62	48-52	870
8	37	19	1600
9	45	51	2150

y = years; Del ex = Deletion exon; Dup ex = Duplication exon; Prom = Promoter; CPK (UI/l) = Creatine kinase (normal value < 190 U/L).

### Cardiovascular magnetic resonance

**Figure 1:**
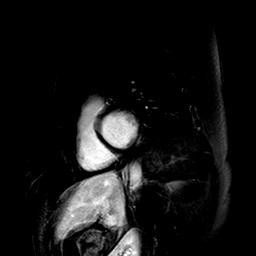
**Myocardial LGE findings in asymptomatic DMDc.** CMR short-axis image of asymptomatic Duchenne carrier (ID 5, 32-year old). Myocardial LGE was only subepicardial and confined to the segments 5 and 6 of the inferolateral left ventricular wall.

**Figure 2:**
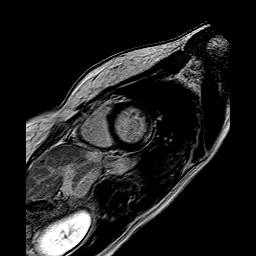
**Midwall fibrosis in asymptomatic DMDc.** CMR short-axis plane of asymptomatic Duchenne carrier (ID 6, 30-year old), representing the midwall fibrosis of the inferolateral left ventricular wall. Both presented patients (ID 5 and ID 6) had normal left ventricular function, without regional hypokinesia in all the segments LGE-positive.

Table 2 shows LGE data in asy and sym carriers: 13 asy-DMDc were LGE negative and 8 were LGE positive. In one young sym-DMDc (ID 1, 20 year-old) who was moderately affected, subepicardial plus mid-miocardial LGE were seen. The average RV and LV EF values were normal, both in DMDc and controls (Table 3). No evidence of myocardial edema was seen in DMDc. LGE positive segments were as follows: 4 basal inferior, 5 basal infero-lateral, 6 basal antero-lateral, 10 mid inferior, 11 mid infero-lateral and 12 mid antero-lateral. Absolute LGE quantity was higher in sym-DMDc than asy-DMDc (1.77 ± 0.27 vs 0.76 ± 0.17 ml, F = 19.6, p < 0.0001). LGE percentage was also higher in sym-DMCc than in asy-DMDc (1.86 ± 0.26 vs 0.68 ± 0.17%, F = 22.1, p < 0.0001) (Table 4). There was no correlation between LGE presence and age. LGE-involved LV segments were most frequently segments 5 and 6. Segment 5 was involved in all LGE positive DMDc. In asy-DMDc (Figure 1) and sym-DMDc, subepicardial LGE was more prevalent than mid-myocardial one. In 5 DMDc (2 asy and 3 sym) (Table 2), LGE was subepicardial plus midmyocardial (Figure 2). There were statistically significant LGE distribution differences in sym-DMDc vs asy-DMDc (Table 4): in the formers, midmyocardial LGE was significantly (0.56 ± 0.09 vs 14 ± 0.05, F = 15.1, p < 0.0001) more frequent than subepicardial LGE. Subendocardial LGE was never seen. RV was LGE negative in all DMDc. All controls were LGE negative in RV and LV.

**Table 2:** LGE data in asymptomatic and symptomatic DMD carriers

**Patient**	**LGE**	**LGEq (ml)**	**LGE (%)**
Asymptomatic			
1	-	0	0
2	-	0	0
3	-	0	0
4	-	0	0
5	+ sepi	2	2
6	+ mid/sepi	3	2,6
7	-	0	0
8	-	0	0
9	-	0	0
10	-	0	0
11	-	0	0
12	-	0	0
13	-	0	0
14	-	0	0
15	+ mid	2	1,6
16	+ sepi	2	2
17	+ sepi	2	1,6
18	+ sepi	2	1,6
19	+ sepi	1	1
20	+ mid/sepi	2	2
21	-	0	0
Symptomatic			
1	+ mid/sepi	1	1
2	-	0	0
3	+ mid	2	2,3
4	+ sepi	3	3
5	-	0	0
6	+ mid	4	4
7	+ mid/sepi	3	3
8	-	0	0
9	+ mid/sepi	3,5	3,5

LGE = late gadolinium enhancement; LGEq (mls) = LGE quantity (milliliters); LGE (%) = LGE percent; + = positive; − = negative; sepi = sub-epicardial; mid = mid-myocardial.

**Table 3:** Clinical, and CMR data in controls and in asymptomatic versus symptomatic Duchenne carriers

**Variables**	**Controls**	**Asymptomatic**	**Symptomatic**	**F**	**p<**
	**(n = 37)**	**(n = 21)**	**(n = 9)**		
**Demographic, clinical**					
Age (months)	418 ± 22	441 ± 29	501 ± 44	1.4	0.25
Body surface area (m^2^)	1.68 ± 0.02	1.65 ± 0.03	1.63 ± 0.04	0.8	0.44
Heart rate (b/min)	78 ± 1	75 ± 1	80 ± 2	2.5	0.09
**CMR**					
Left ventricle (LV)					
Ejection fraction (%)	65 ± 1	63 ± 1	61 ± 2	2.0	0.14
End-diastolic volume (ml/m^2^)	106 ± 3	112 ± 4	109 ± 5	0.8	0.47
End-systolic volume (ml/m^2^)	37 ± 1	42 ± 2	42 ± 3	1.9	0.15
Mass (g)	108 ± 3	97 ± 4	93 ± 6^(*)^	4.3	0.02
Right ventricle(RV)					
Ejection fraction (%)	60 ± 1	60 ± 1	61 ± 2	0.2	0.83
End-diastolic volume (ml/m^2^)	100 ± 3	108 ± 4	98 ± 6	1.4	0.27
End-systolic volume (ml/m^2^)	40 ± 2	43 ± 2	39 ± 3	0.9	0.42

DMDc = Duchenne muscular dystrophy carriers. Data are corrected by BSA and mean ± standard errors.

**Table 4:** CMR data in controls and in asymptomatic versus symptomatic Duchenne carriers

**Variables**	**Controls**	**Asymptomatic**	**Between DMDc p<**	**Symptomatic**	**F**	**p<**
	**(n = 37)**	**(n = 21)**		**(n = 9)**		
LGE quantity (ml)	0	0.76 ± 0.17	0.05	1.77 ± 0.27	19.6	0.0001
Percent LGE versus LV mass (%)	0	0.68 ± 0.17	0.02	1.86 ± 0.26	22.1	0.0001
LGE location						
Sub-endocardial	0	0	-	0	-	
Mid-myocardial	0	0.14 ± 0.05	0.02	0.56 ± 0.09	15.1	0.0001
Sub-epicardial	0	0.33 ± 0.07	0.57	0.44 ± 0.11	10.7	0.0001
Topographic distribution						
Segment 4	0	0.14 ± 0.05	0.08	0.44 ± 0.09	9.8	0.0002
Segment 5	0	0.33 ± 0.07	0.09	0.67 ± 0.11	18.3	0.0001
Segment 6	0	0.29 ± 0.07	0.05	0.67 ± 0.10	18.1	0.0001
Segment 10	0	0.05 ± 0.04	0.15	0.22 ± 0.07	4.5	0.05
Segment 11	0	0.09 ± 0.05	0.11	0.33 ± 0.08	6.9	0.01
Segment 12	0	0.14 ± 0.06	0.24	0.33 ± 0.08	6.2	0.01

DMDc = Duchenne muscular dystrophy carriers. Data are mean ± standard errors.

Dystrophin, genomic analysis and dystrophin gene mutations study. We identified 16 different exons deletions of the dystrophin gene, two duplications and a nonsense mutation (Table 1), in agreement with the known allelic heterogeneity occurring in distrophinopathies [22]. All deletions/duplications were out-of-frame, predicting protein truncations. In two DMDc (ID 4 and 14), a deletion removing the muscle promoter region was identified, nulling transcription of the *in cis* dystrophin allele. The majority of the identified mutations were located in the domain II spectrin-like repeats (exons 10–60, 24 patients), clustered in the proximal rod domain, (exons 10–44, 4 patients). The exon’s study, the mutation type analysis and the affected gene region and protein domain study, did not show correlation between the cardiac phenotype and genotype.

## Discussion and conclusions

This is a cross-sectional study presenting a large group of DMDc in whom, although modest in absolute and relative quantities (Table 4), we observed LGE evidence of myocardial fibrosis in face of normal LV systolic and diastolic functions. Myocardial LGE was not correlated to age and LV function, as the only asy-DMDc with mildly reduced LV EF had no evidence of myocardial fibrosis. LGE quantity and percent were higher in sym-DMDc. Subepicardial plus mid-myocardial LGE were more prevalent in sym-DMDc. LGE pattern preserved the subendocardium and was located subepicardially, at the level of basal infero-lateral wall, most frequently distributed in segment 5 where we speculate that the fibrotic process may start. Our study demonstrates a similar LGE distribution pattern as compared to the study performed by Hor et al. who reported an LGE prevalence of 42.7% in the LV free wall segments of Duchenne patients [23]. Basal mid-myocardial LGE in infero-lateral wall was observed in Becker Xp21-linked muscular dystrophy [24]. Early CMR in acute myocarditis, showed subepicardial or midwall LGE [25] but our results indicate that myocardial LGE was not a consequence of subclinical myocarditis since myocardial edema was not found. CMR is the gold standard to evaluate the presence and location of fibrosis in DMD affected patients [26] and may be performed even in otherwise normal DMDc cardiac phenotype. Myocardial LGE is considered a form of *in vivo* histologic assessment [27] and although limited to brief reports, is increasingly being studied in DMDc, aimed at looking to early signs of cardiac involvement and monitoring disease progression [28,29]. LGE presence in DMDc without LV dysfunction, might be a focal sign of heart involvement either isolated or representing the cardiac evolution of skeletal muscle disease. LGE may be seen in mid-myocardial alone. The midwall fibrosis *per se* might be the result of combined factors including abnormal cardiac energetics and genetic factors [30]. Histopathological corroboration of LGE-CMR abnormalities in earlier stage disease in DMDc remains limited to absent but the advantage might be the possibility to obtain it serially in otherwise normal DMDc cardiac phenotype, aimed at monitoring disease progression.

Some exons’ duplications were more frequently associated with DCM [31]. In our study group, the only DMDc carrying exon’s 2 duplication had subepicardial LGE with normal LV function.

### Study limitations

A major limitation of our CMR technique was that T1 mapping for measuring diffuse myocardial fibrosis [32] was not performed. Current CMR LGE does not detect diffuse microscopic fibrosis. We have assumed that LGE was related to fibrosis, although no histological validation has been performed in our population. Moreover, we did not report data on troponin T to rule out the possible correlation between acute myocarditis and LGE. The cross-sectional nature of this study limits its ability to determine the precise mechanisms or rate of progression of fibrosis, because cause-and-effect cannot be defined. A larger, prospectively followed-up cohort, may give further information on additional causes of fibrosis, its prognostic value, and its potential response to medical therapy if any. In refining LV diastolic and systolic function as causative of myocardial LGE presence, we did not perform Tissue Doppler Imaging (TDI). In this regard, both E/E’ ratio and Sa peak early velocity study, in the setting of normal diastolic and systolic functions, may not be useful in familial cardiomyopathy carriers, before they develop full phenotype [33]. Moreover, neither were data by CMR strain and strain rate measured which may have provided additive insight.

### Clinical implications and strengths

The myocardium in DMDc is a cell milieu where LV dysfunction might be virtually present. We have shown that LGE is frequently found, it is not correlated with age and gene deletion type, and in absolute and relative terms it is modest. However, subepicardial LGE is present yet at an early age. We speculate that when LGE extends from basal infero-lateral subepicardial level to midmyocardium, cardiac involvement might be advanced. Presently, we ignore whether subepicardial or midwall LGE may progress towards other myocardial segments, and how long is needed and/or whether this potential progression may in some way influence DCM development. The association between LGE presence and quantities and the development of LV dysfunction while time passes, remain to be investigated. Based on the present study, the largest conducted thus far, CMR may be useful in DMDc to define the starting LV segment, an information which may not be obtained by ECG, conventional Doppler or 2D-echocardiography.

## Abbreviations

Atl-201-MPS: Adenosine thallium-201 myocardial perfusion scintigraphy; CMR: Cardiovascular magnetic resonance; DCM: Dilated cardiomyopathy; DMDc: Duchenne muscular dystrophy carrier; ECG: Electrocardiography; EF: Ejection fraction; IRGE: Inversion recovery gradient echo; LGE: Late gadolinium enhancement; LV: Left ventricular; MLPA: Multiplex-Ligation Probe Amplification; Pcr/ATP: Phosphocreatine to adenosine triphosphate ratio; RV: Right ventricular; SSFP: Steady-state free precession; TDI: Tissue Doppler imaging.

## Competing interests

The authors declare that they have no competing interests.

## Authors’ contributions

Conception and design: VG and PEP. Drafting of the manuscript: VG, PEP, ER. Analysis and interpretation of data: VG, GC, SW DellaS, SS. Molecular and genetic studies: AF, FG. Critically revision of the manuscript for important intellectual content: VG, PEP, M Di G, ER, GC, GA, FS. All Authors read and approved the final manuscript.
